# Mortality Among Minority Populations with Systemic Lupus Erythematosus, Including Asian and Hispanic/Latino Persons — California, 2007–2017

**DOI:** 10.15585/mmwr.mm7007a2

**Published:** 2021-02-19

**Authors:** Milena A. Gianfrancesco, Maria Dall’Era, Louise B. Murphy, Charles G. Helmick, Jing Li, Stephanie Rush, Laura Trupin, Jinoos Yazdany

**Affiliations:** ^1^Division of Rheumatology, Department of Medicine, School of Medicine, University of California, San Francisco; ^2^Division of Population Health, National Center for Chronic Disease Prevention and Health Promotion, CDC.

Systemic lupus erythematosus (SLE) is a multisystem autoimmune disease with manifestations that vary widely in severity. Although minority populations are at higher risk for SLE and have more severe outcomes (*1*), population-based estimates of mortality by race and ethnicity are often lacking, particularly for Asian and Hispanic/Latino persons. Among 812 patients in the California Lupus Surveillance Project (CLSP) during 2007–2009 (*2*,*3*), who were matched to the 2007–2017 National Death Index (NDI), 16.6% had died by 2017. This proportion included persons of White (14.4%), Black (25%), Asian (15.3%), and Hispanic/Latino (15.5%) race/ethnicity. Standardized mortality ratios (SMRs) of observed-to-expected deaths among persons with SLE within each racial/ethnic group were 2.3, 2.0, 3.8, and 3.9, respectively. These findings provide the first population-based estimates of mortality among Asian and Hispanic/Latino persons with SLE. Coordination of robust care models between primary care providers and rheumatologists could ensure that persons with SLE receive a timely diagnosis and appropriate treatments that might help address SLE-associated mortality.

CLSP included residents of San Francisco County, California, during January 1, 2007–December 31, 2009. Potential patients were identified using community rheumatology and nephrology clinics, community hospitals, and integrated health care systems (*2*,*3*). Clinical information was ascertained through review of medical records.* The State of California Institutional Review Board granted a waiver for this public health surveillance activity, and the project was reviewed and approved by the University of California, San Francisco, Institutional Review Board. This activity was also reviewed by CDC and conducted consistent with applicable federal law and CDC policy.^†^ Patients were not contacted for this linkage study.

Patients with SLE were defined using either the American College of Rheumatology (ACR) classification criteria (at least four of the 11 revised criteria as defined in 1982 and updated in 1997) (*4*,*5*) or two alternative definitions: SLE diagnosed by the patient’s treating rheumatologist plus three ACR criteria or lupus-related kidney disease (World Health Organization class II–VI lupus nephritis upon biopsy or documented record of SLE diagnosis and dialysis or renal transplantation). SLE patients in CLSP were submitted to the 2007–2017 NDI to search for potential matches (general sensitivity of 81.2%–97.9% and specificity of approximately 100%) (*6*). Matching required at least one of the following data items or combinations: first and last name and social security number, first and last name and month and year of birth, social security number, or full date of birth and sex. If none of these combinations was available, the case had insufficient information for submission to NDI and was excluded from analyses. Among the 909 patients with SLE in CLSP, 812 (89%) had sufficient information to be able to be linked with the 2007–2017 NDI. Patients were considered a match based on provided information including social security number.^§^ Multivariable-adjusted risk ratios examining factors associated with mortality were estimated using a Poisson regression model adjusting for age group, sex, race, Hispanic/Latino ethnicity, and number of years since diagnosis. Population estimates by age group, sex, race, and Hispanic/Latino ethnicity for San Francisco County during 2007–2017 were obtained from CDC Wonder^¶^ and were used to calculate SMRs using indirect age standardization in 11 age groups, as the ratio of observed deaths among persons with SLE to expected deaths in the San Francisco County population. Expected deaths were calculated by multiplying the overall age-specific death rate of the general population in San Francisco County by the total number of SLE patients in each age group; age-specific death rates of the general population by sex, race, and ethnicity were also calculated. Two-sided hypothesis tests were conducted controlling for the type I error rate at 5% (α = 0.05) and estimated 95% confidence intervals. Stata (version 16.0; StataCorp) was used to conduct all analyses.

Among the 812 SLE patients analyzed, 731 (90%) were female; race/ethnicities included White (38%), Black (20%), Asian (36%), and Hispanic/Latino (17%), and 5% of persons were of mixed/other race. A total of 135 (16.6%) deaths were identified. Mean age at diagnosis among all SLE patients was 34.9 years (range = 19.0–50.8 years), and mean age at death was 62.0 years (range = 46.2–77.8 years). Mortality increased with age. The highest percentage of deaths (25%) occurred among Black SLE patients; this group had a significantly increased risk for mortality after adjusting for age group, sex, ethnicity, and disease duration (Table 1). On average, Black persons died 6.8 years earlier than did White persons (p = 0.05); persons of Hispanic/Latino ethnicity died 9.5 years earlier than did persons who were not of Hispanic/Latino ethnicity (p = 0.02).

**TABLE 1:** Factors associated with mortality among patients with systemic lupus erythematosus (SLE) — California Lupus Surveillance Project,* 2007–2017

Characteristic	Deaths/No. of SLE patients	% Mortality	Multivariable-adjusted risk ratio^†^ (95% CI)
**Overall**	**135/812**	**16.6**	**NA**
**Sex**			
Female	119/731	16.3	1.0 (0.6–1.6)
Male	16/81	19.8	Reference
**Age group (yrs)**			
10–34	11/204	5.4	Reference
35–44	15/175	8.6	1.5 (0.7–3.1)
45–54	26/185	14.1	2.1 (1.1–4.2)
55–64	33/153	21.6	3.3 (1.8–6.3)
65–74	35/70	50.0	7.4 (4.0–14.0)
≥75	15/25	60.0	9.3 (4.8–18.0)
**Race**			
White	45/312	14.4	Reference
Black	41/164	25.0	1.5 (1.1–2.3)
Asian	45/295	15.3	1.2 (0.8–1.7)
Other	2/22	9.1	1.3 (0.3–5.9)
**Ethnicity^§^**			
Non-Hispanic/Latino	112/604	18.5	Reference
Hispanic/Latino	19/123	15.5	1.1 (0.7–1.8)

**Abbreviations:** CI = confidence interval; NA = not available.

* Population estimates by age group, sex, race, and ethnicity for San Francisco County during 2007–2017 were obtained from CDC Wonder (https://wonder.cdc.gov). Data are from the Multiple Cause of Death Files, 1999–2019 accessed March 21, 2020 (https://wonder.cdc.gov/mcd.html).

^†^ Risk ratios estimated using a multivariable Poisson model that modeled sex, age group, race (White, Black, Asian, other), ethnicity (non-Hispanic/Latino or Hispanic/Latino) simultaneously, adjusting for years since diagnosis. A total of 104 patients were excluded from the multivariable model: 19 (including two deaths) were missing race information, and 85 (including four deaths) were missing Hispanic/Latino ethnicity status.

^§^ Hispanic/Latino ethnicity is considered a distinct concept from race; therefore, it was collected and reported separately from race.

Overall, SMRs were three times higher among SLE patients than among those in the general population of San Francisco County (Table 2). Compared with SMRs among their non-SLE counterparts, SMRs among patients with SLE were four times higher in females and among persons of Asian and Hispanic/Latino race/ethnicity, three times higher among males, and two times higher among White and Black persons. Among females, SMRs were especially high among Asian (4.1) and Hispanic/Latina (5.8) patients.

**TABLE 2:** Age-standardized mortality ratios (SMRs)* for persons with systemic lupus erythematosus (SLE),**^†^** overall and by sex, race,^§^ and Hispanic/Latino ethnicity^¶^—California Lupus Surveillance Project, 2007–2017

Characteristic	No. of SLE patients	No. of observed deaths	No of expected deaths	SMR (95% CI)
**Overall**	**812**	**135**	**45.7**	**3.0 (2.5–3.5)**
**Sex**				
Female	731	119	31.6	3.8 (3.1–4.5)
Male	81	16	5.6	2.9 (1.7–4.7)
**Race**				
White	312	45	20.0	2.3 (1.6–3.0)
Black	164	41	20.8	2.0 (1.4–2.7)
Asian	295	45	12.0	3.8 (2.7–5.0)
**Ethnicity (females and males)**				
Hispanic/Latino	123	19	4.9	3.9 (2.4–6.1)
**Race/Ethnicity** (females)**				
White	283	40	13.8	2.9 (2.1–3.9)
Black	146	37	14.8	2.5 (1.8–3.4)
Asian	264	37	9.0	4.1 (2.9–5.7)
Hispanic/Latina	110	18	3.1	5.8 (3.5–9.2)

**Abbreviation**: CI = confidence interval

* SMR is a ratio between the observed number of deaths in those with SLE and the number of deaths expected, based on age groups defined in CDC Wonder (https://wonder.cdc.gov). Sex, race and Hispanic/Latino ethnicity specific rates in San Francisco County were used, depending on the particular characteristic examined. CIs are calculated for each estimated SMR by assuming a Poisson distribution.

^†^ Age in 2008 was used for adjustment.

**^§^** Forty-one patients were excluded from race-specific analyses, including four who died: 22 had missing race information and 19 identified as a race other than White, Black or Asian, for which estimates are not available through CDC Wonder.

**^¶^** Eighty-five patient records missing Hispanic/Latino ethnicity status, including four deaths, were excluded from ethnicity-specific estimates.

** For female-specific race and ethnicity analyses, crude rates for age group <15 years were not provided by CDC Wonder or were unreliable and therefore not included in calculations; there was insufficient sample size to generate specific race/ethnic estimates for men.

## Discussion

Mortality was almost four times higher than expected among Asian and Hispanic/Latino persons with SLE and was especially high among Hispanic/Latina females. Consistent with other SLE cohorts, Black persons with SLE had higher mortality than did White persons (*7*,*8*). However, this analysis did not find an association between lower mortality and either Asian race or Hispanic/Latino ethnicity, as has been previously reported in the Medicaid population (*7*). Socioeconomic position might interact with race among Medicaid recipients, and there might be potential differences in the non-SLE comparator populations that were included in the other studies. Results of this study are consistent with findings from studies that demonstrated higher mortality among persons with SLE than that in the general population (*1*,*9*); one recent report found a threefold higher SMR among persons with SLE, using a similar study design and methods, though limited to Black and White persons (*8*). There are important gaps in knowledge about SLE among Asian and Hispanic/Latino populations; reasons for these knowledge gaps include smaller sample sizes in observational studies and lower likelihood of Asian and Hispanic/Latino persons being represented in insurance claim data sets (*10*). CLSP provides a unique opportunity to examine SLE incidence, prevalence, and outcomes in these groups because of the relative higher proportion of racial and ethnic populations (Hispanic/Latino, Asian, and Black) among the total population within the area and a comprehensive case finding approach. Mortality for these groups was especially high: Asian females with SLE were four times more likely to die than were Asian females without SLE in the general San Francisco County population, and Hispanic/Latina females with SLE were six times more likely to die than were persons in the corresponding general populations. Higher mortality within these populations might be the result of more severe outcomes and manifestations of SLE, as previously demonstrated (*3*), or possibly less access to care.

The findings in this report are subject to at least five limitations. First, SLE patients might not have been included in the initial CLSP surveillance study unless seen by a specialist. However, this is unlikely given the treatment needs of persons with SLE; further, capture/recapture methods from the initial CLSP study suggested that only two patients were missed (*2*). Second, deaths might not have been identified among the 97 patients with insufficient information to match with NDI. Third, race and ethnicity were determined from the medical record and could be misclassified. Fourth, the number of incident cases (117) and corresponding deaths (23) was small and therefore results could not be provided for incident versus prevalent cases. Finally, results might not be generalizable outside of San Francisco County. The strengths of this study include the use of a comprehensive, population-based surveillance study of well-defined SLE patients, the relatively large numbers of Asian and Hispanic/Latino persons, and the long period for observing mortality.

Mortality among persons with SLE is high among all racial and ethnic groups but is especially pronounced in Asian and Hispanic/Latino populations. CDC,** the Lupus Foundation of America,^††^ and ACR^§§^ are conducting high-impact research investigations to advance the understanding of racial, ethnic, and socioeconomic disparities among persons with SLE, and to develop SLE-specific interventions, such as coordination of robust care models between primary care providers and rheumatologists to ensure that persons with SLE receive a timely diagnosis and appropriate treatments that might help address SLE-associated mortality.

**Summary**

**What is already known about this topic?**

Systemic lupus erythematosus (SLE) is an autoimmune disease that disproportionately affects minority populations. Estimates of SLE mortality by race and ethnicity are lacking, particularly for Asian and Hispanic/Latino persons.

**What is added by this report?**

In a population-based study of SLE patients in San Francisco County during 2007–2017, mortality among Asian persons with SLE was four times higher, and among Hispanic/Latina females with SLE mortality was six times higher, than that of their counterparts in the general population.

**What are the implications for public health practice?**

Coordination of robust care models between primary care providers and rheumatologists could ensure persons with SLE receive a timely diagnosis and appropriate treatments that might help address SLE-associated mortality.

## Footnotes

* Potential patients were identified using the following *International Classification of Diseases, Ninth Revision*, *Clinical Modification* diagnostic codes: 710.0 (SLE), 695.4 (discoid lupus), 710.8 (other specified connective tissue disease), and 710.9 (unspecified connective tissue disease). Secondary sources of possible patients included a commercial laboratory, which was queried for a comprehensive panel of SLE-related serologic tests and the California Office of Statewide Health Planning and Development hospital discharge database (https://doi.org/10.1002/art.40191).

† 45 C.F.R. part 46.102(l)(2), 21 C.F.R. part 56; 42 U.S.C. Sect. 241(d); 5 U.S.C. Sect. 552a; 44 U.S.C. Sect. 3501 et seq.

§ Except for one case in which the social security number was not included in the NDI record but was considered a match through first and last name, month and year of birth, and state where death took place.

¶
https://wonder.cdc.gov

**
https://www.cdc.gov/lupus/

††
https://www.lupus.org/

§§
https://www.rheumatology.org/
